# Silent soft tissue pathology is common with a modern metal-on-metal hip arthroplasty: Early detection with routine metal artifact-reduction MRI scanning

**DOI:** 10.3109/17453674.2011.579518

**Published:** 2011-07-08

**Authors:** Henry Wynn-Jones, Rory Macnair, James Wimhurst, Nish Chirodian, Brian Derbyshire, Andoni Toms, John Cahir

**Affiliations:** ^1^The Centre for Hip Surgery, Wrightington Hospital, Lancashire; ^2^Department of Trauma and Orthopaedics, Norfolk and Norwich University Hospital NHS Foundation Hospital, Norwich, Norfolk, UK

## Abstract

**Background and purpose:**

Adverse reactions to metal debris have been reported to be a cause of pain in metal-on-metal hip arthroplasty. We assessed the incidence of both symptomatic and asymptomatic adverse reactions in a consecutive series of patients with a modern large-head metal-on-metal hip arthroplasty.

**Methods:**

We studied the early clinical results and results of routine metal artifact-reduction MRI screening in a series of 79 large-head metal-on-metal hip arthroplasties (ASR; DePuy, Leeds, UK) in 68 patients. 75 hips were MRI scanned at mean 31 (12–52) months after surgery.

**Results:**

27 of 75 hips had MRI-detected metal debris-related abnormalities, of which 5 were mild, 18 moderate, and 4 severe. 8 of these hips have been revised, 6 of which were revised for an adverse reaction to metal debris, diagnosed preoperatively with MRI and confirmed histologically. The mean Oxford hip score (OHS) for the whole cohort was 21. It was mean 23 for patients with no MRI-based evidence of adverse reactions and 19 for those with adverse reactions detected by MRI. 6 of 12 patients with a best possible OHS of 12 had MRI-based evidence of an adverse reaction.

**Interpretation:**

We have found a high early revision rate with a modern, large-head metal-on-metal hip arthroplasty. MRI-detected adverse rections to metal debris was common and often clinically “silent”. We recommend that patients with this implant should be closely followed up and undergo routine metal artifact-reduction MRI screening.

Metal-on-metal (MoM) total hip replacements have been used since the 1960s. Failure in early designs was attributed to mechanical loosening caused by poor bearing tolerances producing high friction (Amstutz and Grigoris 1996, Kothari et al. 1996). Improved manufacturing and engineering techniques enabled the development of a new generation of MoM hip replacements. In the 1990s, the Birmingham Hip Resurfacing (BHR) was developed, and good early to medium-term results have been published (Daniel et al. 2004, Treacy et al. 2005, Heilpern et al. 2008). Similar implants, both resurfacings and large MoM bearings, coupled with standard femoral stems were subsequently developed and marketed by other manufacturers.

The development of magnetic resonance imaging (MRI) metal artifact reduction (MAR) sequences has enabled good visualization of the periprosthetic tissues (Toms et al. 2008), and been reported to be a clinically useful part of the assessment of painful MoM hip replacements (Hart et al. 2009). A number of authors have described the appearance of collections of fluid and inflammatory masses around painful MoM hip arthroplasties (Boardman et al. 2006, Pandit et al. 2008, Toms et al. 2008). These have been grouped under a variety of headings such as “aseptic lymphocyte-dominated vasculitis-associated lesions” (Willert et al. 2005), “pseudotumors” (Pandit et al. 2008), or “adverse reactions to metal debris (ARMD)” (Langton et al. 2010). Although these lesions have been previously described in patients investigated for pain, there have been no studies on the overall incidence of these lesions in an unselected series of patients, including those with no, or few, symptoms. It is not known whether these lesions may occur in the absence of symptoms.

At our institution, we have a policy of offering routine MAR MRI imaging to patients who have undergone MoM total hip replacement or resurfacing. We determined the early clinical outcome, revision rate, and incidence of ARMD using MAR MRI screening in a consecutive series of patients with an ASR THR or resurfacing (ASR; DePuy, Leeds, UK).

## Patients and methods

The ASR system was used at our institution between February 2005 and March 2008. This study is a report of the results of our standard follow-up and imaging protocol. 79 hip arthroplasties using ASR components were performed in 68 patients by 5 surgeons. 17 ASR resurfacing procedures were performed in 14 patients. 62 THRs were performed in 54 patients using an ASR acetabular component, a matched cobalt-chrome ASR XL head, and a Corail titanium hydroxyapatite-coated uncemented stem (DePuy, Leeds, UK). 14 head sizes were available, ranging from 39 mm to 63 mm in diameter in 2-mm increments. For the purposes of comparative analysis, we designated femoral head component sizes in the range 39–49 mm as “small”, and 51–63 mm as “large”.

The mean age of the 79 cases (56 males) at the time of surgery was 55 (30–76) years. The mean time from the primary procedure to last follow-up or revision was 32 (14–51) months. No patients had died or were lost to follow-up. Indications for surgery were primary osteoarthritis (OA) (n = 70), OA secondary to dysplasia (n = 3), post trauma (n = 2), avascular necrosis (n = 2), and OA secondary to Perthes' (n = 2).

### Implants

The median size of the femoral head component was 49 (43–57) mm). For the resurfacing group, the median size was 51 (45–57) mm and for the THR group it was 49 (43–55) mm. 31 cases had a “large” femoral head (51–63 mm) and 48 cases a “small” head (38–49 mm).

### Follow-up assessments

The departmental policy at our institution is that all patients who have undergone a MoM hip replacement should remain under review and be assessed annually. The review involves clinical assessment, radiological assessment, and a patient-based self-assessment questionnaire. The questionnaire includes the Oxford hip score (Dawson et al. 1996) (OHS) (where 12 = best score and 60 = worst score), an assessment of the patients' satisfaction with the outcome of their hip replacement (possible responses: Yes, No, Uncertain), and rating the result of their hip replacement on a visual analog scale (VAS) from 0 (unsatisfactory) to 10 (perfect).

Patients who were scheduled for revision were asked to complete a questionnaire before to revision surgery. All patients at our hospital with a MoM hip replacement are also routinely invited to undergo an MRI scan, even if they are asymptomatic (provided there are no contraindications).

### Plain radiographs

Plain radiographs were assessed by one of the authors (HWJ) on a diagnostic PACS workstation. The acetabular implant orientation, leg length, offset, and femoral component alignment were measured. Two techniques were used for measurement of acetabular component orientation. Acetabular inclination angle was measured manually (on the earliest postoperative, anteroposterior, supine pelvis radiograph of sufficient quality) with reference to the inter-teardrop line using tools on the PACS workstation. Acetabular component orientation was also measured using Wrightington cup orientation software. This enables measurement of inclination and version, and corrects for angular artifact due to the central X-ray beam offset from the hip (Derbyshire and Porter 2003, Derbyshire 2008). 2 of the authors (HWJ and BD) tested the inter-observer and intra-observer reliability of this software using standard statistical techniques (Bland and Altman 1983, Ranstam et al. 2000). Using these measurements, we designated acetabular components as being within or outside Lewinnek's “safe zone” (anteversion 5–25 degrees, and inclination 30–50 degrees) (Lewinnek et al. 1978).

Serial radiographs were compared to assess for periprosthetic osteolysis, lucent lines, bone loss, prosthesis migration, and soft tissue swelling. We noted osteolysis and radiolucent lines greater than 1 mm around the acetabular component in the zones of DeLee and Charnley (DeLee and Charnley 1976) as modified by Beaulé et al. (2004). Radiolucency around the femoral stem was recorded using the zones of Amstutz et al. (2004) for resurfacing arthroplasty and of Gruen for stemmed total hip arthroplasty.

### Metal artifact-reduction MRI

All MR examinations were performed on a 1.5T machine (Siemens Symphony; Siemens Healthcare, Erlangen, Germany) using sequences adapted for metal artifact suppression. All images were reviewed by two musculoskeletal radiologists (each had 5 years' experience in reporting MRI findings around MoM hip prostheses of various designs) and consensus findings were recorded.

**Figure 1.**
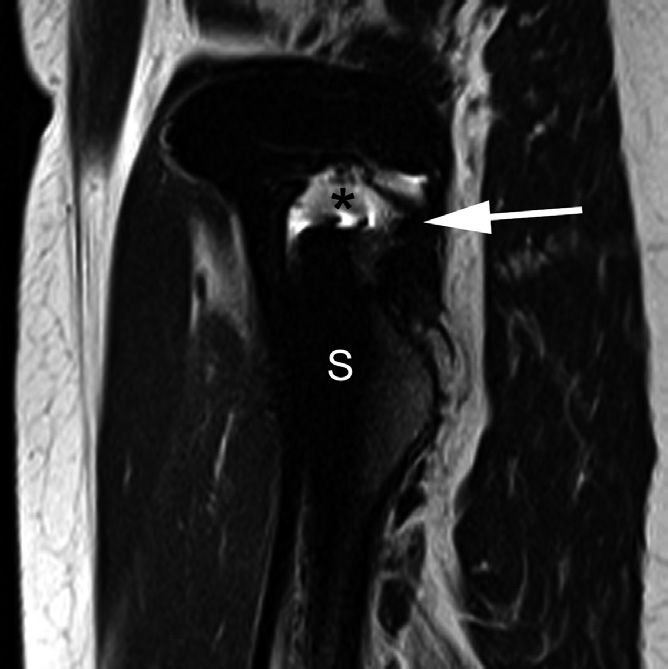
Mild adverse reaction to metal debris. Sagittal T2W MR through the femoral stem (S) of a Corail total hip replacement demonstrating mild periprosthetic disease. A small fluid-filled cavity (asterisk) surrounding the neck of the prosthesis is encapsulated by a thick, ragged low-signal rim (white arrow).

**Figure 2.**
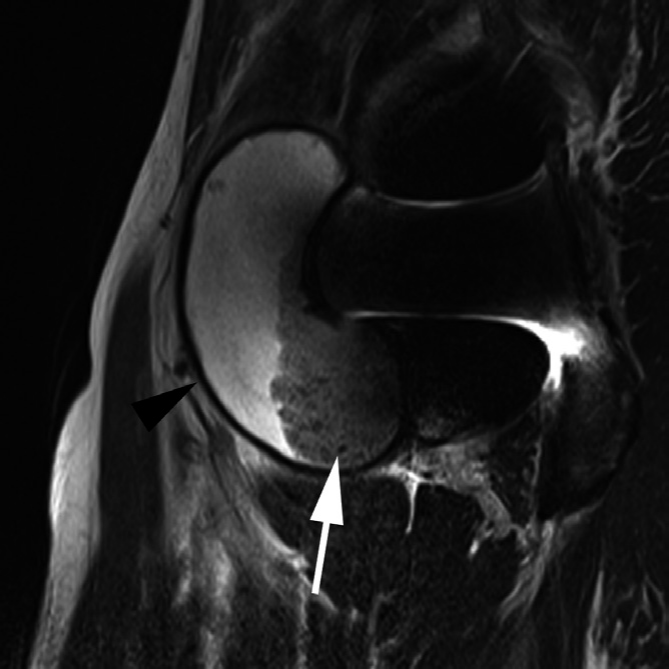
Moderate adverse reaction to metal debris. A sagittal T2W MR positioned just medial to the acetabular cup demonstrates moderate periprosthetic disease with a large cystic collection, demarcated by a low signal wall (black arrow), and filled with debris (white arrow) extending proximally in the line of the iliopsoas bursa. The relatively thick low signal wall and the debris are not typical of conventional iliopsoas bursae.

**Figure 3.**
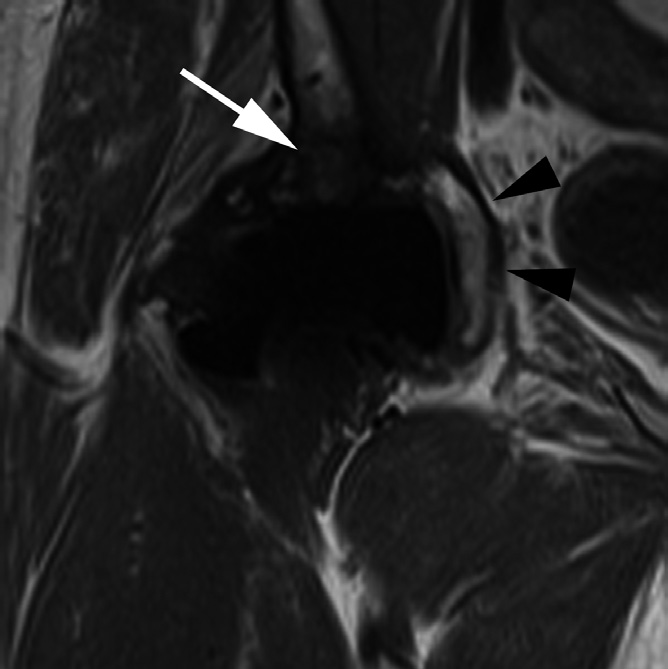
Severe adverse reaction to metal debris. Coronal T1W MR through the mid-coronal plane of the femoral head (black arrows indicate the medial wall of the acetabulum), demonstrating severe periprosthetic disease with bone marrow replacement in the acetabular roof (white arrow).

Findings were categorized as: normal (Cahir et al. 2007), abnormal and typical of an adverse reaction to metal debris (Fang et al. 2008, Pandit et al. 2008, Toms et al. 2009), or abnormal but typical of a disease other than a metal-on-metal reaction—e.g. infection (Cahir et al. 2007). For those cases with characteristic findings of ARMD, they were further classified into mild, moderate, or severe disease (Figures 1–3). Mild changes constituted periprosthetic collections less than 5 cm in diameter, moderate comprised soft tissue masses of fluid collections greater than 5 cm in diameter, gluteal muscle atrophy or bone marrow edema and severe changes including extension through deep fascia, tendon avulsion, bone marrow replacement or fracture, or neurovascular involvement. This grading system has been shown to be reliable (Anderson et al. 2011).

### Histopathology

The tissue specimens in those patients who were revised, or underwent a biopsy, were assessed by a histopathologist experienced in evaluation of metal debris-related periprosthetic tissue reactions.

### Statistics

Statistical analysis was performed using StatsDirect statistical software version 2.7.7 (StatDirect Ltd., Altrincham, UK). Dichotomous variables were analyzed using Fisher's exact test. Continuous parametric data were analyzed using unpaired t-tests and non-parametric data were assessed with the Mann-Whitney U-test.

## Results

### Plain radiographs

The mean cup inclination angle measured manually on digital plain radiographs was 50 (36–74) degrees. The acetabular orientation using Wrightington cup orientation software had a mean inclination of 50 (34–75) degrees and mean anteversion of 12 (2.3–39) degrees.

The intra- and inter-observer reliabilities of Wrightington cup orientation software for measuring ASR acetabular orientation were satisfactory. The intra-observer repeatability for version was ± 0.55 degrees, and it was ± 0.49 degrees for inclination. The inter-observer limits of agreement (95%) for version were –1.9 to 6.6 degrees, and for inclination they were –2.8 to 2.4 degrees. All 79 acetabular components appeared to be well fixed, with good bone ongrowth on the last follow-up radiograph. None of the acetabular components had osteolysis or radiolucent lines greater than 1 mm in any of the three Charnley DeLee zones.

16 of the 17 resurfacing femoral components had no evidence of loosening, migration, neck thinning, or radiolucent lines around the stem in any of Amstutz zones. One patient had neck thinning, with resorption of the superior aspect of the femoral neck on the anteroposterior radiograph. There was no lysis, and no radiolucent lines around the stem of the femoral resurfacing component.

52 of the 62 Corail stems appeared well fixed on the latest radiographs, and had no radiolucent lines in any of the 7 Gruen zones. 10 hips had radiolucent lines in 1 or more Gruen zone. In 7 of these hips, a radiolucent line was seen only in Gruen zone 1. 3 hips also had lucent lines in Gruen zone 7. In all 10 hips, the Corail femoral component appeared well fixed from zones 2 to 6, and had not migrated.

### MAR MRI examinations

75 patients had MAR MRI examinations (59 THR, 16 resurfacing) at a mean of 31 (12–52) months after surgery. 4 hips were not scanned because 2 patients (3 hips) had a contraindication to MRI (1 pacemaker and 1 spinal cord stimulator) and 1 patient declined to be scanned as he was claustrophobic (Table 1). 42 MRI scans were classified as consistent with normal postoperative appearances (including seromas and atrophy of the short external rotators). 33 scans were considered to be abnormal, of which 3 were not thought to be typical of an adverse reaction to metal debris, including: infection (n = 1), iliopsoas bursa (n = 1), and osteolysis (n = 1). 27 scans were considered to be abnormal and demonstrated features consistent with an adverse reaction to metal debris. 5 cases were considered to be mild, 18 were considered moderate, and 4 were classified as severe. The typical appearance was of a fluid signal collection extending from, and surrounding, the bearing that was demarcated by a very low-signal capsule, which was often ragged. Debris and a heterogeneous signal were common findings within the fluid collections. The patients with severe disease included 3 cases with bone marrow replacement around either the acetabulum (n = 1) or the proximal femur (n = 2), and 1 patient had encasement of the sciatic nerve. The radiologists were not able to classify 3 of the MRI examinations and recommended follow-up with repeat imaging after a further 6 months. There were 2 cases of atrophy of the gluteus medius and minimus but no cases of gluteal avulsion. There were 7 cases of bone marrow edema in the proximal femur without any other abnormal findings. The significance of bone marrow edema in the proximal femur is unknown, but may be part of the spectrum of normal MAR MRI appearances in the absence of other changes. These cases were classified as normal postoperative appearances.

**Table 1.** Summary of metal artifact-reduction (MAR) MRI findings

	MAR MRI findings								
A	B	C	D	E	F	G	H	I	J
ASR resurfacing	7	0	2	3	1	6	3	1	17
Corail with XL head and ASR cup	35	3	3	15	3	21	0	3	62
Total	42	3	5	18	4	27	3	4	79

A Prosthesis B Normal C Abnormal, not ARDM D Mild ARMD E Moderate ARMD F Severe ARMD G All ARDM H Unclassifiable I Not scanned J Total

In patients with normal MAR MRI findings or abnormalities that were not an adverse reaction, the mean corrected cup inclination angle was 50 (34–75) degrees, the mean anteversion was 13 (2.3–33) degrees, and mean head size was 50 (43–57) mm. In those patients with an adverse reaction to metal debris, the mean cup inclination was 50 (37–60) degrees, mean anteversion was 12 (2.3–39) degrees, and mean head size was 49 (45–57) mm. The MAR MRI findings, and potential risk factors associated with ARMD, including sex, head size (large: > 50 mm, small: < 50 mm) and acetabular orientation (inclination, version, and location within or outside Lewinneck's “safe zone”(Lewinnek et al. 1978)) are summarized in Table 2. There was an increased risk of MRI-detected adverse reaction to metal debris with small femoral heads and cup orientation outside Lewinnek's “safe zone”, but this increase was not statistically significant.

**Table 2.** Metal artifact-reduction MRI findings in relation to potential risk factors for metal debris-related reactions

	MRI classification		
	Not ARMD	ARMD	p-value
	(A and B)	(C1, C2, C3)	
Sex, F / M	13 / 35	9 / 18	0.4
Mean head size (mm)	50	49	0.2
Mean acetabular inclination (°)	50	51	0.4
Mean acetabular anteversion (°)	13	12	0.2
Head, small / large	26 / 22	19 / 8	0.2
Cup in Lewinnek's “safe zone”, yes / no	24 / 21	10 / 16	0.2

### Implant survival

At a mean follow-up of 32 (14–51) months, 8 revisions had been performed in 8 patients (4 female) (Table 3). The cumulative revision rate at 40 months with revision for any reason was 11% (95% CI: 4–18).

**Table 3.** Summary of revisions

A	B	C	D	E	F	G	H	I	J	K
1	51	F	P	34	30	47	58	20	+	+
2	59	M	P	40	40	49	49	24	+	+
3	60	M	S	34	15	51	60	13	+	+
4	55	F	P	38	46	51	56	13	+	–
5	57	F	P	32	14	51	57	3	(+)	+
6	67	F	P+S	23	22	45	53 **^a^**	**^a^**	+	+
7	60	M	P	16	57	49	56	11	–	–
8	55	M	P	29	55	47	53	7	+	+

**^a^** Only conventional radiograph available; cup inclination measured manually. A Case B Age C Sex D Indication for revision P pain S squeaking E Time to revision (months) F OHS G Head size (mm) H Cup inclination I Cup version J MRI findings – normal (+) mild + moderate K Histology (+ ARMD)

All the cases that were revised had an ASR acetabular component with an XL head and a Corail femoral component. 6 revisions were performed for pain (1 of these patients also reported squeaking). MRI confirmed an adverse reaction to metal debris before revision in 4 of these patients. 2 patients had minimal pain (1 had a squeaking hip), but screening MRI revealed changes consistent with a moderate adverse reaction to metal debris in one case and mild in the other case that was squeaking. Both patients elected to undergo revision. The plain radiographs were unremarkable, with no osteolysis in 7 of these patients. In 1 patient, there were radiolucencies with the appearance of a neocortex in Gruen zones 1 and 7. At the time of revision, the proximal stem was found to be loose with necrotic tissue, metal-stained debris, and fluid between the stem and the bone. The stem was well fixed distally. This patient underwent revision of both components. In the remaining revisions, the femoral component was preserved and the acetabular component revised to an uncemented acetabular component with a polyethylene or ceramic liner. The XL heads were exchanged to appropriate ceramic heads to match the acetabular components.

### Histopathology

All 6 patients with a MAR MRI diagnosis of ARMD had histopathological findings (in the tissue taken at the time of revision) consistent with an ARMD. The findings were similar in the 6 cases: a fibrous capsular wall was seen, showing fibrinoid proliferation, with surface necrosis. A wide band of bland necrosis was seen. Perivascular lymphocytic infiltration was seen with macrophages or histiocytes containing small metal particles.

The histology in the 2 patients who were revised for pain, but with normal MRI scans, revealed normal fibrous tissue with no evidence of inflammation or adverse reaction to metal debris.

### Patient-related outcome

All hips were assessed with a hip questionnaire and an OHS at mean 32 (14–51) months after the primary procedure. The assessment scores were those at the latest follow-up or last assessment prior to revision.

66 patients were satisfied with their hip replacements whereas 7 were not, and 6 were doubtful. The mean overall “success” rating by patients of their hip replacements (on a VAS from 1 to 10) was 8.

The mean OHS in all patients—either at the latest follow-up or before revision—was 21. In the 8 patients who had a revision, the mean OHS before revision was 37. In patients without MRI-based evidence of an adverse reaction to metal debris, the mean OHS was 23, and in those with MRI-based evidence of ARMD it was 19 (p = 0.3) (Table 4).

**Table 4.** Patient-related outcome in relation to MAR MRI findings

A	B	C	D	E	F	G
Normal	42	33	5	4	7.9	23
Abnormal (not ARMD)	3	3	0	0	8	25
Mild ARMD	5	5	0	0	9	18
Moderate ARMD	18	14	1	2	7.6	21
Severe ARMD	4	4	0	0	9.5	13
Unclassifiable	3	3	0	0	9	15
Not scanned	4	4	0	0	9.5	12
All	79	66	6	6	8.2	21

A MRI classification B No. of cases Patient satisfaction: C Yes D Doubtful E No F VAS (0–10): 0 = unsatisfactory; 10 = perfect. G OHS: 12 = best; 60 = worst.

51 patients had an OHS at latest follow-up of 20 or less, and 18 of these patients had MRI-based evidence of an adverse reaction to metal debris. 26 patients had a “perfect” OHS of 12 at latest follow-up, and 6 of these had MRI-based evidence of ARMD.

## Discussion

We found MRI-detected metal debris-related abnormalities in one third of patients with a modern MoM bearing. Previous studies have concentrated on the MRI findings in patients investigated for painful prostheses (Boardman et al. 2006, Fang et al. 2008, Pandit et al. 2008, Toms et al. 2008).

One of our most concerning findings was that MRI-based evidence of an adverse reation to metal debris does not appear to correlate with symptoms. In fact, some of the highest levels of satisfaction were in those patients with the worst MAR MRI findings. One quarter of patients with a best possible OHS (12) had MRI-based evidence of ARMD. This suggests that even a policy of frequent clinical review would not detect patients developing soft tissue complications until extensive damage had occurred. It is unclear why there is often no pain.

A comparison can be made with the problem of silent osteolysis, which is well documented in patients with uncemented acetabular components with a polyethylene liner (Hozack et al. 1996, Utting et al. 2008). It is generally accepted that patients with such implants should be routinely assessed from plain radiographs—even in the absence of symptoms—in order to detect osteolysis before it becomes extensive. The difference with metal-on-metal related pathology is that soft tissue pathology is of particular concern, and this is not visible on a plain radiograph. We believe it is preferable to detect ARMD soft tissue damage and fluid-filled cavities at an early stage before the damage becomes extensive and irreversible. Grammatopolous et al. (2009) reported that resurfacing prostheses revised for pseudotumors have a poor outcome. This may well be because, in their series, patients only presented once they had become symptomatic and the disease had become extensive. Our experience with an earlier-generation 28-mm bearing MoM prosthesis, used in the 1990s, was that it functioned well for several years and then some patients suddenly presented with severe extensive soft tissue and bone necrosis, which was often undetectable on plain radiographs (Nolan et al. 2008).

The pattern of disease seen in our series on MRI shares similarities with those previously described for other prostheses, but there are also key differences. The pseudocysts in this group of patients commonly contained debris resulting in heterogeneous signal patterns (Figure 2), whereas those described with other prostheses were typically homogeneous fluid-filled cavities (Fang et al. 2008, Toms et al. 2008, 2009). Gluteal myositis, atrophy, and avulsion have been described on MR with metal-on-metal-associated disease (Toms et al. 2008) but these were not common findings in our series of patients. This may be because MRI has been performed on asymptomatic patients and patients earlier in their postoperative course than previously described.

A number of factors, including female sex, small prosthetic head size, “poor” acetabular component orientation, and component design may contribute to ARMD. Hart et al. (2009) have shown that in a series of 16 failed large-head MoM prostheses, 13 were positioned outside the Lewinnek “safe zone”. We have found MRI-based evidence of MoM disease in 41% of prostheses with “small heads” (38–49 mm) and cup orientations outside Lewinneck's “safe zone”. With “large heads” and a cup within the “safe zone”, the incidence of MoM disease was still one fifth. Lewinnek's safe zone originally related to dislocation risk in metal-on-polyethylene hip replacements. There have been no prospective studies of acetabular component position to confirm whether there is actually a safe zone for prevention of ARMD. It is possible that all MoM prostheses, in any orientation, would develop a reaction.

Our cumulative revision rate at 40 months of 11% is much higher than that for a conventional THR. In our series, the overall revision rate for ARMD was 8%. Other authors have reported a high early revision rate with the ASR. Langton et al. (2010) reported poor early results with the ASR system, with a revision rate for symptomatic ARMD of 3% at 3 years. The revision rate in the subgroup of patients in their series with a total hip replacement (ASR cup, ASR XL head, and a Corail stem) rather than resurfacing was higher, at 6%. The Australian National Joint Replacement Registry 2009 report (AOA NJRR 2009) found that the number of revisions per 100 observed component years for the ASR was 2.3 as compared to 0.8 for the Birmingham Hip Resurfacing (BHR).

The design of the ASR acetabular component may be one of the reasons for the high failure rate. The cup comprises between 148 and 160 degrees of a sphere, whereas the BHR ranges from 158 to 166 degrees. This means that for any given cup position, more of the ASR head is uncovered. This may lead to increased edge loading and wear of the ASR cup. In a comparative study of the ASR and BHR, Langton et al. (2009) found that serum chromium and cobalt levels from ASR prostheses were more strongly influenced by the effect of the orientation of the acetabular component. There was an increase in metal ions at inclinations > 45° and anteversion angles of < 10° and > 20° with the ASR, whereas these levels were only increased in the BHR group when the acetabular components were implanted at inclinations > 55°.

We conclude that in our series of patients, the ASR Corail THR had a high rate of early revision due to MoM-related soft tissue problems. Furthermore, the incidence of MRI-detected MoM disease was high in both ASR Corail THRs and ASR resurfacings. Many patients with lesions revealed by MAR MRI were asymptomatic. We recommend that all patients with this implant be carefully followed up on a regular basis. We believe that routine assessment of these implants should include soft tissue imaging.
